# Genetic variant rs17225178 in the *ARNT2* gene is associated with Asperger Syndrome

**DOI:** 10.1186/s13229-015-0009-0

**Published:** 2015-02-27

**Authors:** Agnese Di Napoli, Varun Warrier, Simon Baron-Cohen, Bhismadev Chakrabarti

**Affiliations:** Department of Psychiatry, Autism Research Centre, University of Cambridge, Douglas House, 18B Trumpington Road, Cambridge, CB2 8AH UK; Cambridgeshire and Peterborough NHS Foundation Trust, CLASS Clinic, Cambridge, UK; Centre for Integrative Neuroscience and Neurodynamics, School of Psychology and Clinical Language Sciences, University of Reading, Reading, UK

**Keywords:** Autism spectrum conditions (ASC), Asperger Syndrome (AS), Aryl-hydrocarbon receptor nuclear translocator 2 (*ARNT2*), Single nucleotide polymorphisms (SNPs)

## Abstract

**Background:**

Autism Spectrum Conditions (ASC) are neurodevelopmental conditions characterized by difficulties in communication and social interaction, alongside unusually repetitive behaviours and narrow interests. Asperger Syndrome (AS) is one subgroup of ASC and differs from classic autism in that in AS there is no language or general cognitive delay. Genetic, epigenetic and environmental factors are implicated in ASC and genes involved in neural connectivity and neurodevelopment are good candidates for studying the susceptibility to ASC. The aryl-hydrocarbon receptor nuclear translocator 2 (*ARNT2*) gene encodes a transcription factor involved in neurodevelopmental processes, neuronal connectivity and cellular responses to hypoxia. A mutation in this gene has been identified in individuals with ASC and single nucleotide polymorphisms (SNPs) have been nominally associated with AS and autistic traits in previous studies.

**Methods:**

In this study, we tested 34 SNPs in *ARNT2* for association with AS in 118 cases and 412 controls of Caucasian origin. *P* values were adjusted for multiple comparisons, and linkage disequilibrium (LD) among the SNPs analysed was calculated in our sample. Finally, SNP annotation allowed functional and structural analyses of the genetic variants in *ARNT2*. We tested the replicability of our result using the genome-wide association studies (GWAS) database of the Psychiatric Genomics Consortium (PGC).

**Results:**

We report statistically significant association of rs17225178 with AS. This SNP modifies transcription factor binding sites and regions that regulate the chromatin state in neural cell lines. It is also included in a LD block in our sample, alongside other genetic variants that alter chromatin regulatory regions in neural cells.

**Conclusions:**

These findings demonstrate that rs17225178 in the *ARNT2* gene is associated with AS and support previous studies that pointed out an involvement of this gene in the predisposition to ASC.

## Background

Autism Spectrum Conditions (ASC) are a group of neurodevelopmental conditions defined by difficulties in communication and social interaction, alongside unusually repetitive behaviours and narrow interests. Asperger Syndrome (AS) is a subtype of these conditions, where there is no delay in language or cognitive development [1]. The incidence of ASC in the general population is approximately 1 in 100 [2]. Male:female sex ratios of 9:1 and 4:1 are observed in individuals with AS [3] and classic autism [4], respectively, although the true sex ratio in AS may be closer to the sex ratio in classic autism because of under-detection of AS in females [5]. ASC are heritable [6,7] and multifactorial conditions, where genetic, epigenetic and environmental factors are involved [8,9].

ASC are neurodevelopmental in origin, and accordingly, genes involved in neural development and connectivity are good candidate genes for studying the genetics of these conditions [10,11]. The aryl-hydrocarbon receptor nuclear translocator 2 (*ARNT2*) gene encodes a transcription factor of the basic helix-loop-helix (bHLH)-Per-ARNT-Sim (PAS) family, which is mainly expressed in the kidney and central nervous system [12,13]. ARNT2 dimerizes with the hypoxia-inducible factor-1α (HIF-1α) and regulates cellular responses to hypoxia [14]. It also dimerizes with single-minded homolog 1 (SIM1) and participates in the development of paraventricular and supraoptic nuclei in mice [15]. Variants in the *SIM1* gene have been associated with obesity in humans and have been shown to reduce transcriptional activity of SIM1 combined with ARNT2. Interestingly, 11 individuals carrying these variants have reported atypical behaviours, such as ASC [16]. SIM1 controls the expression of methyl CpG binding protein 2 (*MECP2*) gene, which is disrupted in individuals with Rett Syndrome who display autistic features [17]. *Mecp2* mouse models show typical features of Rett Syndrome [18,19], and knockout mice with a deletion of *Mecp2* in *Sim1*-expressing neurons display altered social behaviour [20]. Heterozygous *Arnt2* mutant mice lack secretory neurons in paraventricular and supraoptic nuclei, implicating the involvement of ARNT2 in neurodevelopmental processes [21]. Arnt2 and Sim1 are also involved in long-range axonal guidance and hence lay down the connections between the hypothalamus and the spinal cord in zebrafish [22]. ARNT2 is a transcription factor in mouse hippocampus [23] and regulates the brain-derived neurotrophic factor (*Bdnf*) gene that controls neural activity and is related to several human neurodevelopmental conditions [24]. A recent study by Lin and colleagues proposed a role of *ARNT2* in the predisposition to ASC. They tested the levels of expression of candidate genes for ASC in neural aggregates in human stem cell models (comparable to a telencephalon in the early stages of development) after heat shock and they observed an alteration of *ARNT2* expression [25]. One study reported a missense mutation in *ARNT2* in individuals with ASC [26] and a previous study from our laboratory identified nominal associations between single nucleotide polymorphisms (SNPs) rs3901896 and rs4778599 with AS and autistic traits, respectively [27]. A recent study has reported nominally significant association for SNPs in *ARNT2* and *SIM1* with autistic traits in a general population sample [28].

In the current study, we tested 34 SNPs in *ARNT2* for association with AS in an independent sample, composed of 118 cases and 412 controls. We found a significant association of rs17225178 with AS after correcting for multiple comparisons and a nominal association of rs3848173 with AS. To test the replicability of this observation, we analysed genome-wide association studies (GWAS) data from the Psychiatric Genomics Consortium (PGC), which tested SNPs for association with ASC in case–control and family-based samples [29]. Our study provides further support for a role of *ARNT2* in ASC and in particular it points out an involvement of a SNP (that is rs17225178) in the predisposition to AS.

## Methods

### Participants

Participants were recruited by advertisement from the volunteer database of the Autism Research Centre in Cambridge (United Kingdom). They included both individuals with an AS diagnosis and individuals who were not diagnosed with AS and did not have any close family member with a diagnosis of ASC. All individuals completed the Autism Spectrum Quotient (AQ) online [30] to separate cases and controls from the two extreme ends of the AQ continuum. Eighty percent of individuals with an ASC diagnosis score above 32 on the AQ [31]. Cases were previously diagnosed by independent clinicians in the United Kingdom for having AS using the International Statistical Classification of Diseases and Related Health Problems, Tenth Revision (ICD-10) [32] or the Diagnostic and Statistical Manual of Mental Disorders, Fourth Edition, Text Revision (DSM-IV-TR) [1] criteria, at recognized clinics in the United Kingdom, by trained psychiatrists or clinical psychologists. A proportion of these were diagnosed at our clinic in Cambridge (the CLASS Clinic). Individuals without an ASC diagnosis and with an AQ score below or equal to 24 were selected as controls. This ensured that individuals with high autistic traits but without a diagnosis of ASC (the so-called “Broader Autism Phenotype” (BAP) [33]) were not included in the study, as this can confound the analysis. The AQ measures autistic traits, has high heritability [34] and has a normal distribution in the general population [30]. Individuals in the general population show a mean AQ score of 16.4 ± 6.3, while individuals with AS have a mean AQ score of 35.8 ± 6.5 [31]. All participants resided in the United Kingdom when the study was conducted and reported Caucasian ancestry for at least three generations. Overall, we tested 530 individuals, among them 118 cases (74 males and 44 females) with a mean AQ score of 35.6 ± 8.9 (males mean: 35.1 ± 8.7, females mean: 36.6 ± 8.8) and 412 controls (185 males and 227 females) with a mean AQ score of 14.9 ± 5.0 (males mean: 16.0 ± 4.4, females mean: 13.9 ± 5.1) (Table 1).

**Table 1:** **Schematic description of the participants enrolled in the current study**

**Status of the participants**	**Total number**	**Number of males**	**Number of females**	**Total AQ score**	**AQ score in males**	**AQ score in females**
Cases	118	74	44	35.6 ± 8.9	35.1 ± 8.7	36.6 ± 8.8
Controls	412	185	227	14.9 ± 5.0	16.0 ± 4.4	13.9 ± 5.1

Total numbers of participants and total AQ scores for cases and controls are reported. These values stratified by sex are also reported. AQ, Autism Spectrum Quotient.

### SNP selection and genotyping

Thirty-four SNPs in the *ARNT2* gene were selected from 80704652 bp to 80889940 bp on chromosome 15 with a mean inter-SNP distance of 7 Kb (GRCH37.p10 Primary Assembly, National Center for Biotechnology Information [NCBI]). We did not have a specific hypothesis about any SNP in particular and tested multiple SNPs within the same gene to identify a possible association of the gene with a given phenotype [35]. All chosen SNPs had a minor allele frequency (MAF) > 0.05 in the HapMap CEU (Utah residents with northern and western European ancestry) population (HapMap genome browser, release 27) and were available in the TaqMan SNP Genotyping Assays (Applied Biosystems Inc., Foster City, CA, USA), that was used for genotyping in the current study. rs1446336, rs8034535 and rs7175825 are tag SNPs, as indicated by the HapMap genome browser (release 27). The most upstream SNP is rs1446336 (Chr 15: 80704652) and the most downstream is rs1139650 (Chr 15: 80889940) (see Table 2). Buccal samples were collected by mail from the participants and DNA was extracted and anonymized [36]. SNP genotyping was performed following the protocol previously reported [27]. Hardy-Weinberg Equilibrium (HWE) was tested in controls using Plink v1.07 [37] at *α* = 0.05, and all selected SNPs were in HWE.

**Table 2:** **SNPs in** ***ARNT2*** **analysed in the current study**

**SNP ID**	**Chromosomal position**	**Major**/**minor allele**	**MAF (CEU)**
rs1446336	80704652	T/A	0.407
rs16972073	80709303	G/C	0.075
rs12594558	80709766	C/T	0.259
rs1026016	80710955	C/T	0.153
rs3910982	80712122	C/A	0.333
rs1020397	80718738	G/C	0.283
rs4778790	80721271	A/G	0.389
rs1912	80723361	C/T	0.138
rs8034535	80725974	A/G	0.208
rs8036233	80729687	T/A	0.474
rs3901896	80734097	C/T	0.394
rs3848173	80734588	C/T	0.129
rs17788120	80742881	T/C	0.411
rs17225178	80743866	T/A	0.081
rs3848175	80747103	G/A	0.281
rs895444	80749761	A/G	0.111
rs12591546	80754740	G/T	0.275
rs4778795	80760069	G/T	0.226
rs12905523	80760688	T/C	0.240
rs4778798	80770223	T/C	0.042
rs7181179	80778586	T/C	0.009
rs4778599	80781763	G/A	0.296
rs7175825	80795419	G/C	0.442
rs11858186	80801472	C/T	0.491
rs12439920	80804741	G/A	0.103
rs11072922	80806183	C/T	0.159
rs4778604	80837819	C/T	0.492
rs4423382	80854992	A/C	0.155
rs10851935	80859935	T/C	0.341
rs7403706	80861365	T/C	0.168
rs7403013	80874261	G/A	0.127
rs4072568	80884025	G/A	0.137
rs6495511	80888783	A/G	0.243
rs1139650	80889940	A/G	0.093

The table shows SNP ID and chromosomal position of genetic variants analysed in this study. Major/minor allele and MAF values in the CEU population for each SNP are also reported. CEU, Utah residents with northern and western European ancestry; MAF, minor allele frequency; SNP, single nucleotide polymorphism.

### Statistical analysis

The Chi-Square statistics was applied to test association between 34 SNPs in *ARNT2* and AS, using Plink v1.07. We used Bonferroni correction to adjust *P* values at the *α* threshold of significance (*α* = 0.05). Since Bonferroni correction is conservative when the tests are not independent, we used the SNPSpD web interface [38], to account for linkage disequilibrium (LD) patterns between all SNPs in the current sample. The effective number of independent loci was 21 and the subsequent threshold of significance after Bonferroni correction was *α* = 0.0024. We analysed LD blocks in our sample using Haploview [39].

Functional annotation of significant SNPs was conducted using the following softwares: HaploReg v2 [40] allows analysis of non-coding genetic variants in terms of their effect on the organization of chromatin regions and regulatory motifs; F-SNP [41] is a tool which combines multiple databases for providing the functional roles of genetic variants; the Genetic Association Database [42] gives information about genetic studies which previously identified association between the SNPs we analysed and ASC; SNPnexus [43] is an online database which was used to define regulatory elements and conserved regions; the University of California Santa Cruz (UCSC) genome browser [44] permits to validate functions of the SNPs analysed.

### Analysis of replication data

We had access to the summary data of the PGC database and we checked for effect sizes and *P* values for rs17225178 in the ASC cohort. The PGC analysed genome-wide SNPs for association with ASC in 161 cases, 526 controls, 4,788 trio cases and 4,788 trio pseudocontrols all of Caucasian ancestry [29]. Details of methods, ancestry of the participants and analyses for this cohort are provided in reference [29].

### Consent and ethics

Participants gave their informed consent before participating in this study. The current study had ethics approval from the Cambridge Psychology Research Ethics Committee and the NHS Research Ethics Committee (UK) and was conducted in accordance with the principles of the Declaration of Helsinki.

## Results

rs17225178 was significantly associated with AS in our sample with *P* value = 0.001, and rs3848173 was nominally associated with AS in our sample. Minor alleles of these two SNPs are more prevalent in cases than in controls with odds ratio (OR) > 1 and are associated with an increased risk of AS (Table 3). To replicate our association, we searched for the *P* value and effect direction of rs17225178 in the PGC dataset. rs17225178 was significant (*P* value = 0.0121), and the effect direction was same between the two datasets (OR > 1) (Table 3).

**Table 3:** **Results of SNP association analyses in our sample and in the PGC database**

**SNP ID**	**MAF (cases)**	**MAF (controls)**	**SNP association analysis in our sample**			**SNP association analysis in the PGC database**		
			**OR**	***X*** ^**2**^	***P*** **value**	**OR**	**SE**	***P*** **value**
rs3848173	0.2288	0.1667	1.484	4.768	0.029			
rs17225178	0.1966	0.1173	1.842	9.773	***0.001771***	1.115	0.0433	0.0121

Nominally significant *P* values after genetic association analysis between 34 SNPs in *ARNT2* and AS in our sample are reported (*α* = 0.05). Significant *P* value after Bonferroni correction for total number of SNPs is written in bold and italicized (*α* = 0.0024). Major allele is the reference allele for the estimated OR. Results of genetic association analysis between rs17225178 and ASC in the PGC database are also reported. AS, Asperger Syndrome; ASC, Autism Spectrum Conditions; MAF, minor allele frequency; OR, odds ratio; PGC, Psychiatric Genomics Consortium; SE, standard error for the odds ratio; SNP, single nucleotide polymorphism.

**Figure 1:**
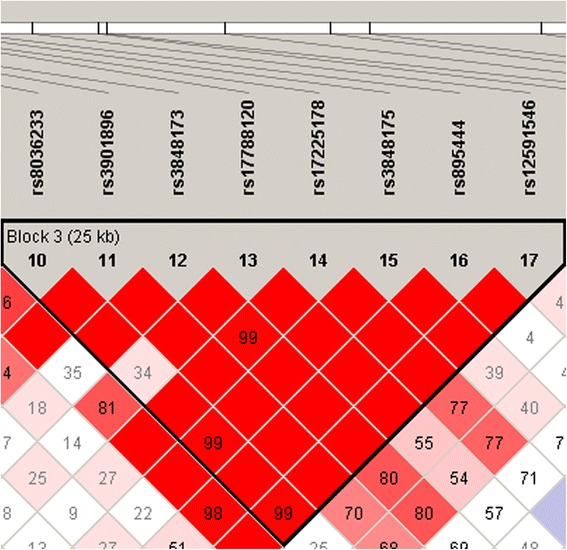
**Linkage disequilibrium blocks in the** ***ARNT2*** **gene calculated in our sample (detail).** Block 3 is shown. It includes eight SNPs analysed in the current study. Numbers into the squares indicate *D*’ values.

**Figure 2:**
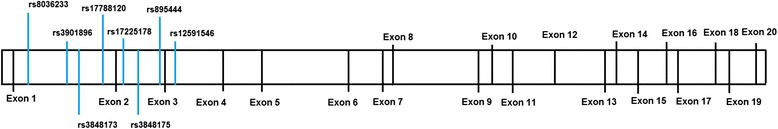
**Schematic diagram of the** ***ARNT2*** **gene.** White boxes indicate introns. SNP significantly associated with AS in the current study and those in linkage disequilibrium with this genetic variant in our sample are indicated by blue lines.

rs17225178 is included in a LD block (25 Kb) in our sample, alongside seven other SNPs (rs3848173, rs8036233, rs3901896, rs17788120, rs3848175, rs895444 and rs12591546) (Figure 1). rs17225178 is located in intron 2 of *ARNT2*, alongside rs3848175 and rs895444. Intron 1 includes rs8036233, rs3901896, rs3848173 and rs17788120; while rs12591546 is located in intron 3 (Figure 2).

## Discussion

The aim of this study was to investigate the role of common genetic variants in the predisposition to AS, which is a high-functioning form of ASC. The complex genetic nature of ASC has led previous studies to examine multiple genes, mainly involved in neural development and connectivity, sex-hormones signaling and social-emotional pathways [10,11,27]. In the current study, we tested for a genetic association between 34 SNPs in *ARNT2* with AS, and then replicated our significant SNP in a large ASC cohort. Our results show nominal association of rs3848173 with AS and significant association between rs17225178 and AS. We replicated the association of rs17225178 in the PGC ASC cohort, corroborating previous reports that showed an involvement of *ARNT2* in ASC. This is consistent with a previous study from our laboratory which indicated nominal association of rs3901896 with AS, and of rs4778599 with autistic traits, measured using the AQ [27]. Recent studies have reported a mutation in the *ARNT2* gene in ASC [26] and common variants associated with autistic traits [28].

*ARNT2* is located on chromosome 15q24 and includes 20 exons and 19 introns (Figure 2). It spans 193.587 Kb and has eight splice variants. Four transcript variants are protein-coding: one is composed of the first 20 exons and 19 introns; two transcripts include the first 19 exons and 18 introns; one transcript includes the first 6 exons and 5 introns (UCSC genome browser). rs17225178 is located in intron 2 (Figure 2) and modifies transcription factor binding sites (TATA, GATA and TAL1 motifs) and genomic regions that regulate the chromatin state in neural cell lines (hippocampus and brain angular gyrus) (HaploReg v2). SNP annotation did not indicate the presence of miRNA binding sites or other structural variants at the level of this SNP and did not provide information about the effect of rs17225178 on protein structure and function (F-SNP, SNPnexus). We hypothesize that the presence of the risk allele of rs17225178 could influence the binding of different transcription factors or could allow the modification of chromatin states, regulating chromatin accessibility and dynamics. This genetic variant is included in a LD block in our sample, where seven other SNPs (rs8036233, rs3901896, rs3848173, rs17788120, rs3848175, rs895444 and rs12591546) that alter chromatin regulatory regions in neural cell lines are located (HaploReg v2). Two previous studies have reported nominal association of rs3901896 with AS [27] and autistic traits (for example, language impairment) [28]. This genetic variant is in LD with rs17225178 in our sample (Figure 1). An interesting next step will be to perform molecular studies to analyse possible chromatin modifications and binding of specific transcription factors at the level of regulatory regions of *ARNT2*, carrying the risk allele or LD region.

This is the first study that shows a statistically significant association of rs17225178 with AS and supports previous findings that indicate an involvement of *ARNT2* in ASC. This is consistent with the role of *ARNT2* in neural development, as is known from animal models. In particular, *ARNT2* is involved in the development of oxytocinergic neurons [15]. The oxytocinergic system has been implicated in autism through multiple lines of evidence [45-51]. This result provides further support for the atypical oxytocinergic system in ASC by implicating a gene involved in the development of oxytocinergic neurons. In our study, we find a significant association with rs17225178, which is included in a LD block, alongside rs3901896 that has been nominally associated with AS [27] and autistic traits [28]. In the previous genetic study carried out in our laboratory [27], we did not find association between rs17225178 and AS, as we performed SNP association analysis at the gene level and not for each SNP independently.

The limitation of this study is the small sample size, so further research is required to corroborate these results. Animal models of Rett Syndrome have established a role for *Mecp2* [18-20], and it will be important to further test if *Arnt2* play an important role in underlying the autistic phenotype. Future work in animals should employ well-designed behavioural assays relevant to autism in *Arnt2* knockout mice, such as social-preference paradigms [52]. In humans, larger association studies of *ARNT2* SNPs with endophenotypes linked to autism will be particularly relevant.

## Conclusions

In the current study, we report a statistically significant association between the genetic variant rs17225178 in *ARNT2* and AS. Using the PGC ASC dataset, we were able to replicate our results in a larger cohort. The direction of effect was the same in both the cohorts. Our findings provide evidence for the role of *ARNT2* in ASC and show the involvement of rs17225178 in this gene in AS, a subgroup of ASC.

## Abbreviations

AQ: Autism Spectrum Quotient
ARNT2: aryl-hydrocarbon receptor nuclear translocator 2
AS: Asperger Syndrome
ASC: Autism Spectrum Conditions
BAP: broader autism phenotype
BDNF: brain-derived neurotrophic factor
bHLH-PAS: basic helix-loop-helix-Per-Arnt-Sim
CEU: Utah residents with northern and western European ancestry
DSM-IV-TR: Diagnostic and Statistical Manual of Mental Disorders, Fourth Edition, Text Revision
GWAS: genome-wide association studies
HIF-1α: hypoxia-inducible factor-1α
HWE: Hardy-Weinberg Equilibrium
ICD-10: International Statistical Classification of Diseases and Related Health Problems, Tenth Revision
LD: linkage disequilibrium
MAF: minor allele frequency
MECP2: methyl CpG binding protein 2
NCBI: National Center for Biotechnology Information
OR: odds ratio
PGC: Psychiatric Genomics Consortium
SIM1: single-minded homolog 1
SNP: single nucleotide polymorphism
UCSC: University of California Santa Cruz
